# (*E*)-*N*′-(5-Bromo-2-methoxy­benzyl­idene)-4-chloro­benzohydrazide

**DOI:** 10.1107/S1600536808017911

**Published:** 2008-06-19

**Authors:** Hong-Wei Lin

**Affiliations:** a Department of Chemistry, Huaihua University, Huaihua 418008, People’s Republic of China

## Abstract

The title Schiff base compound, C_15_H_12_BrClN_2_O_2_, crystallizes with two independent mol­ecules in the asymmetric unit. The mol­ecules adopt an *E* configuration with respect to the C=N double bond. The dihedral angles between the benzene rings are 24.4 (2) and 9.4 (2)° in the two mol­ecules. The crystal structure is stabilized by inter­molecular N—H⋯O hydrogen bonds, forming chains running along the *b* axis.

## Related literature

For general background, see: Ali *et al.* (2005 ▶); Arıcı *et al.* (2005 ▶); Hebbachi & Benali-Cherif (2005 ▶); Kurtoglu & Ispir (2007 ▶); Qi *et al.* (2007 ▶); Sallam (2007 ▶); Salmon *et al.* (2005 ▶); Sarı *et al.* (2006 ▶); Tuncel & Sari (2007 ▶). For related structures, see: Lin (2007 ▶); Tang (2007 ▶, 2008 ▶); Yang *et al.* (2008 ▶).
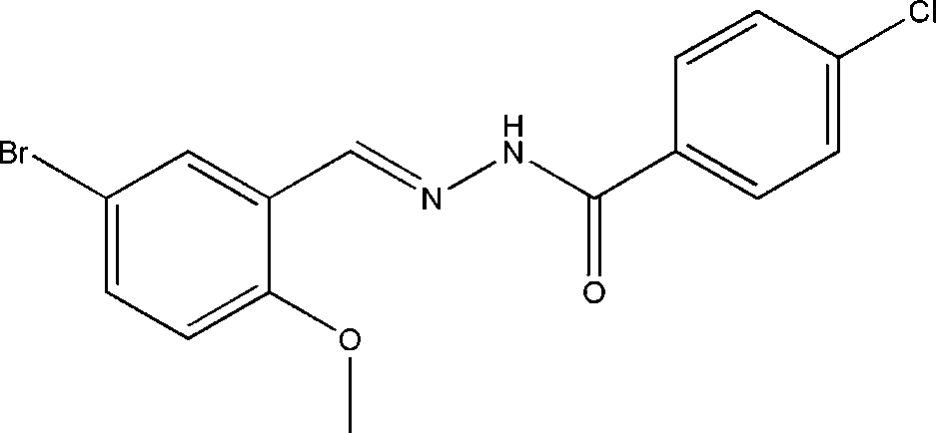


## Experimental

### 

#### Crystal data

C_15_H_12_BrClN_2_O_2_
*M* *_r_* = 367.63
Triclinic, 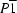

*a* = 7.636 (3) Å
*b* = 9.837 (4) Å
*c* = 20.524 (8) Å
α = 82.045 (5)°
β = 83.660 (6)°
γ = 87.573 (5)°
*V* = 1516.9 (10) Å^3^
*Z* = 4
Mo *K*α radiation
μ = 2.89 mm^−1^
*T* = 298 (2) K
0.20 × 0.20 × 0.18 mm

#### Data collection

Bruker SMART APEX CCD area-detector diffractometer
Absorption correction: multi-scan (*SADABS*; Bruker, 2000 ▶) *T*_min_ = 0.596, *T*_max_ = 0.624
8876 measured reflections
6405 independent reflections
3536 reflections with *I* > 2σ(*I*)
*R*_int_ = 0.027

#### Refinement

*R*[*F* ^2^ > 2σ(*F* ^2^)] = 0.050
*wR*(*F* ^2^) = 0.141
*S* = 1.02
6405 reflections
387 parameters
2 restraints
H atoms treated by a mixture of independent and constrained refinement
Δρ_max_ = 0.53 e Å^−3^
Δρ_min_ = −0.64 e Å^−3^

### 

Data collection: *SMART* (Bruker, 2000 ▶); cell refinement: *SAINT* (Bruker, 2000 ▶); data reduction: *SAINT*; program(s) used to solve structure: *SHELXTL* (Sheldrick, 2008 ▶); program(s) used to refine structure: *SHELXTL*; molecular graphics: *SHELXTL*; software used to prepare material for publication: *SHELXTL*.

## Supplementary Material

Crystal structure: contains datablocks global, I. DOI: 10.1107/S1600536808017911/sj2515sup1.cif

Structure factors: contains datablocks I. DOI: 10.1107/S1600536808017911/sj2515Isup2.hkl

Additional supplementary materials:  crystallographic information; 3D view; checkCIF report

## Figures and Tables

**Table 1:** Hydrogen-bond geometry (Å, °)

*D*—H⋯*A*	*D*—H	H⋯*A*	*D*⋯*A*	*D*—H⋯*A*
N2—H2⋯O4^i^	0.894 (10)	2.026 (16)	2.900 (4)	165 (4)
N4—H4*A*⋯O2^ii^	0.893 (10)	1.994 (18)	2.854 (4)	161 (4)

Symmetry codes: (i) 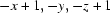; (ii) 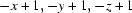.

**Fig. 1.**
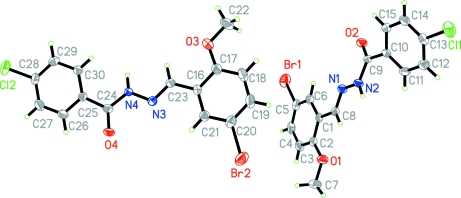
The asymmetric unit of (I) with displacement ellipsoids drawn at the 30% probability level.

**Fig. 2.**
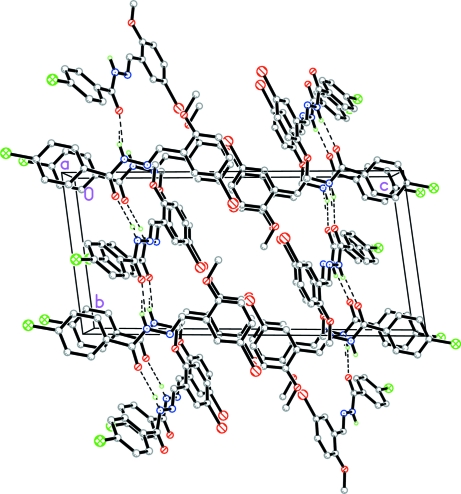
Molecular packing of (I) with hydrogen bonds drawn as dashed lines.

## supplementary crystallographic information

### Comment

Schiff bases are very important ligands as they can readily form stable complexes with most metal ions (Tuncel & Sari, 2007; Sallam, 2007; Salmon *et al.*, 2005; Ali *et al.*, 2005; Arıcı *et al.*, 2005; Hebbachi & Benali-Cherif, 2005; Sarı *et al.*, 2006). Furthermore, Schiff bases and their metal complexes have excellent biological properties (Kurtoglu & Ispir, 2007; Qi *et al.*, 2007). The author has proeviously reported the crystal structure of the Schiff base compound, isonicotinic acid [1-(3,5-dibromo-2-hydroxyphenyl)methylidene]hydrazide methanol solvate, which shows antibacterial activity (Lin, 2007). As a continuation of work on such compounds, I report herein the crystal structure of the new Schiff base compound, (I), Figure 1.

The two unique molecules of (I) adopt *trans* configurations about the C═N double bonds. The bond lengths and bond angles are within normal ranges and comparable to those observed in other similar Schiff bases (Tang, 2007, 2008; Yang *et al.*, 2008). The C8–N1 and C23–N3 bond lengths are respectively 1.268 (4) and 1.281 (5) Å, indicating they are double bonds while the C9–N2 and C24–N4 distances are 1.352 (5) and 1.349 (5) Å respectively, indicating some degree of conjugation in the molecules. The dihedral angle between the C1—C6 and C10—C15 benzene rings is 24.4 (2) ° with a 9.4 (2) ° angle between the  C16—C21 and C25—C30 rings. The crystal structure is stabilized by intermolecular N–H···O hydrogen bonds (Table 1), forming chains running along the *b* axis (Figure 2).

### Experimental

5-Bromo-2-methoxybenzaldehyde (21.5 mg, 0.1 mmol) and 4-chlorobenzohydrazide (17.0 mg, 0.1 mmol) were dissolved in MeOH (10 ml). The mixture was stirred at room temperature for 5 min to give a colorless solution. Colorless needle-like crystals of (I) were obtained from this solution on standing.

### Refinement

Atoms H2 attached to N2 and H4A attached to N4 were located in a difference Fourier map and refined isotropically, with N–H distances restrained to be 0.90 (1) Å. Other H atoms were placed in the calculated positions and constrained to ride on their parent atoms, with C–H distances in the range 0.93–0.96 Å, and with *U*_iso_(H) values set to 1.2*U*_eq_(C) and 1.5*U*_eq_(methyl C).  Crystals of (I) were small and very weakly diffracting reducing the amount of data collected.

### Crystal data

**Table:**

C_15_H_12_BrClN_2_O_2_	*Z* = 4
*M**_r_* = 367.63	*F*_000_ = 736
Triclinic, *P*1	*D*_x_ = 1.610 Mg m^−^^3^
Hall symbol: -P 1	Mo *K*α radiation λ = 0.71073 Å
*a* = 7.636 (3) Å	Cell parameters from 2115 reflections
*b* = 9.837 (4) Å	θ = 2.4–24.5º
*c* = 20.524 (8) Å	µ = 2.89 mm^−^^1^
α = 82.045 (5)º	*T* = 298 (2) K
β = 83.660 (6)º	Cut from a needle, colorless
γ = 87.573 (5)º	0.20 × 0.20 × 0.18 mm
*V* = 1516.9 (10) Å^3^	

### Data collection

**Table:**

Bruker SMART APEX CCD area-detector diffractometer	6405 independent reflections
Radiation source: fine-focus sealed tube	3536 reflections with *I* > 2σ(*I*)
Monochromator: graphite	*R*_int_ = 0.027
*T* = 298(2) K	θ_max_ = 27.0º
ω scans	θ_min_ = 2.0º
Absorption correction: multi-scan(SADABS; Bruker, 2000)	*h* = −9→8
*T*_min_ = 0.596, *T*_max_ = 0.624	*k* = −12→12
8876 measured reflections	*l* = −22→26

### Refinement

**Table:**

Refinement on *F*^2^	Secondary atom site location: difference Fourier map
Least-squares matrix: full	Hydrogen site location: inferred from neighbouring sites
*R*[*F*^2^ > 2σ(*F*^2^)] = 0.050	H atoms treated by a mixture of independent and constrained refinement
*wR*(*F*^2^) = 0.141	*w* = 1/[σ^2^(*F*_o_^2^) + (0.0616*P*)^2^ + 0.0769*P*] where *P* = (*F*_o_^2^ + 2*F*_c_^2^)/3
*S* = 1.02	(Δ/σ)_max_ = 0.001
6405 reflections	Δρ_max_ = 0.53 e Å^−^^3^
387 parameters	Δρ_min_ = −0.64 e Å^−^^3^
2 restraints	Extinction correction: none
Primary atom site location: structure-invariant direct methods	

### Special details

**Table:**

Geometry. All e.s.d.'s (except the e.s.d. in the dihedral angle between two l.s. planes) are estimated using the full covariance matrix. The cell e.s.d.'s are taken into account individually in the estimation of e.s.d.'s in distances, angles and torsion angles; correlations between e.s.d.'s in cell parameters are only used when they are defined by crystal symmetry. An approximate (isotropic) treatment of cell e.s.d.'s is used for estimating e.s.d.'s involving l.s. planes.
Refinement. Refinement of *F*^2^ against ALL reflections. The weighted *R*-factor *wR* and goodness of fit *S* are based on *F*^2^, conventional *R*-factors *R* are based on *F*, with *F* set to zero for negative *F*^2^. The threshold expression of *F*^2^ > σ(*F*^2^) is used only for calculating *R*-factors(gt) *etc*. and is not relevant to the choice of reflections for refinement. *R*-factors based on *F*^2^ are statistically about twice as large as those based on *F*, and *R*- factors based on ALL data will be even larger.

### Fractional atomic coordinates and isotropic or equivalent isotropic displacement parameters (Å^2^)

**Table:**

	*x*	*y*	*z*	*U*_iso_*/*U*_eq_	
Br1	−0.26007 (7)	0.58987 (6)	0.37080 (3)	0.0799 (2)	
Br2	0.70280 (8)	−0.20259 (6)	0.50498 (3)	0.0802 (2)	
Cl1	1.33371 (15)	0.53500 (15)	0.06070 (7)	0.0791 (4)	
Cl2	0.17724 (17)	0.08183 (14)	1.11711 (6)	0.0720 (4)	
O1	0.0684 (3)	0.0824 (3)	0.26908 (15)	0.0538 (8)	
O2	0.5295 (4)	0.6554 (3)	0.20845 (15)	0.0540 (8)	
O3	0.7291 (4)	0.3334 (3)	0.61136 (16)	0.0647 (9)	
O4	0.3751 (4)	−0.1646 (3)	0.83186 (14)	0.0564 (8)	
N1	0.3455 (4)	0.4277 (3)	0.24230 (16)	0.0396 (8)	
N2	0.5140 (4)	0.4259 (3)	0.21166 (16)	0.0381 (8)	
N3	0.5078 (4)	0.0226 (3)	0.73272 (16)	0.0391 (8)	
N4	0.4450 (4)	0.0552 (3)	0.79436 (15)	0.0366 (7)	
C1	0.0856 (5)	0.3112 (4)	0.28578 (18)	0.0393 (9)	
C2	−0.0160 (5)	0.1942 (4)	0.29267 (19)	0.0428 (10)	
C3	−0.1897 (5)	0.1970 (5)	0.3207 (2)	0.0530 (11)	
H3	−0.2577	0.1198	0.3235	0.064*	
C4	−0.2615 (6)	0.3127 (5)	0.3442 (2)	0.0562 (12)	
H4	−0.3773	0.3139	0.3637	0.067*	
C5	−0.1607 (6)	0.4277 (4)	0.3388 (2)	0.0491 (11)	
C6	0.0089 (5)	0.4277 (4)	0.3099 (2)	0.0450 (10)	
H6	0.0742	0.5066	0.3063	0.054*	
C7	−0.0205 (6)	−0.0445 (4)	0.2809 (3)	0.0658 (14)	
H7A	−0.0422	−0.0726	0.3277	0.099*	
H7B	0.0514	−0.1132	0.2609	0.099*	
H7C	−0.1306	−0.0331	0.2620	0.099*	
C8	0.2686 (5)	0.3138 (4)	0.25389 (18)	0.0386 (9)	
H8	0.3261	0.2338	0.2426	0.046*	
C9	0.5984 (5)	0.5460 (4)	0.19608 (19)	0.0387 (9)	
C10	0.7809 (5)	0.5382 (4)	0.16255 (19)	0.0380 (9)	
C11	0.8303 (6)	0.4472 (4)	0.1180 (2)	0.0543 (12)	
H11	0.7496	0.3855	0.1094	0.065*	
C12	1.0006 (6)	0.4474 (4)	0.0860 (2)	0.0619 (13)	
H12	1.0331	0.3881	0.0549	0.074*	
C13	1.1199 (5)	0.5355 (4)	0.1006 (2)	0.0503 (11)	
C14	1.0735 (6)	0.6255 (4)	0.1447 (2)	0.0519 (11)	
H14	1.1560	0.6848	0.1542	0.062*	
C15	0.9042 (5)	0.6280 (4)	0.1750 (2)	0.0470 (10)	
H15	0.8718	0.6909	0.2044	0.056*	
C16	0.6500 (5)	0.1030 (4)	0.62592 (19)	0.0388 (9)	
C17	0.7266 (5)	0.2153 (4)	0.5841 (2)	0.0480 (10)	
C18	0.7957 (6)	0.1997 (5)	0.5206 (2)	0.0634 (13)	
H18	0.8470	0.2738	0.4931	0.076*	
C19	0.7896 (6)	0.0768 (5)	0.4976 (2)	0.0650 (13)	
H19	0.8363	0.0673	0.4546	0.078*	
C20	0.7141 (5)	−0.0335 (5)	0.5381 (2)	0.0530 (11)	
C21	0.6451 (5)	−0.0205 (4)	0.6024 (2)	0.0455 (10)	
H21	0.5954	−0.0957	0.6295	0.055*	
C22	0.7650 (8)	0.4582 (5)	0.5692 (3)	0.0902 (19)	
H22A	0.8828	0.4539	0.5477	0.135*	
H22B	0.7535	0.5332	0.5949	0.135*	
H22C	0.6829	0.4721	0.5365	0.135*	
C23	0.5758 (5)	0.1230 (4)	0.69281 (19)	0.0390 (9)	
H23	0.5779	0.2091	0.7065	0.047*	
C24	0.3841 (5)	−0.0456 (4)	0.8418 (2)	0.0394 (9)	
C25	0.3300 (5)	−0.0040 (4)	0.90879 (19)	0.0356 (9)	
C26	0.2364 (5)	−0.0993 (4)	0.9552 (2)	0.0505 (11)	
H26	0.2062	−0.1820	0.9429	0.061*	
C27	0.1892 (6)	−0.0726 (5)	1.0180 (2)	0.0565 (12)	
H27	0.1274	−0.1369	1.0485	0.068*	
C28	0.2331 (5)	0.0499 (4)	1.0362 (2)	0.0463 (10)	
C29	0.3231 (5)	0.1459 (4)	0.9917 (2)	0.0514 (11)	
H29	0.3519	0.2287	1.0044	0.062*	
C30	0.3706 (5)	0.1186 (4)	0.9278 (2)	0.0455 (10)	
H30	0.4307	0.1838	0.8973	0.055*	
H2	0.563 (5)	0.345 (2)	0.203 (2)	0.080*	
H4A	0.440 (6)	0.1421 (19)	0.803 (2)	0.080*	

### Atomic displacement parameters (Å^2^)

**Table:**

	*U*^11^	*U*^22^	*U*^33^	*U*^12^	*U*^13^	*U*^23^
Br1	0.0826 (4)	0.0853 (4)	0.0695 (4)	0.0282 (3)	0.0066 (3)	−0.0234 (3)
Br2	0.1092 (5)	0.0691 (4)	0.0665 (4)	0.0134 (3)	−0.0071 (3)	−0.0312 (3)
Cl1	0.0522 (7)	0.1017 (10)	0.0773 (10)	−0.0086 (7)	0.0133 (6)	−0.0042 (8)
Cl2	0.0843 (9)	0.0966 (10)	0.0361 (7)	0.0092 (7)	−0.0019 (6)	−0.0191 (6)
O1	0.0520 (17)	0.0475 (17)	0.063 (2)	−0.0136 (14)	0.0019 (14)	−0.0123 (15)
O2	0.0693 (19)	0.0282 (15)	0.062 (2)	−0.0032 (14)	0.0134 (15)	−0.0118 (14)
O3	0.089 (2)	0.0479 (19)	0.053 (2)	−0.0178 (16)	−0.0013 (16)	0.0053 (16)
O4	0.089 (2)	0.0272 (15)	0.0509 (19)	−0.0125 (14)	0.0168 (15)	−0.0122 (13)
N1	0.0406 (19)	0.0320 (18)	0.045 (2)	−0.0011 (14)	0.0018 (15)	−0.0057 (15)
N2	0.0409 (19)	0.0267 (17)	0.045 (2)	−0.0022 (14)	0.0031 (15)	−0.0049 (15)
N3	0.0456 (19)	0.0354 (17)	0.0360 (19)	−0.0010 (15)	0.0004 (15)	−0.0079 (15)
N4	0.0527 (19)	0.0285 (16)	0.0277 (18)	−0.0052 (15)	0.0038 (14)	−0.0055 (15)
C1	0.044 (2)	0.046 (2)	0.026 (2)	−0.0040 (19)	0.0002 (17)	−0.0007 (18)
C2	0.050 (2)	0.049 (3)	0.030 (2)	−0.004 (2)	−0.0038 (18)	−0.0053 (19)
C3	0.047 (3)	0.063 (3)	0.046 (3)	−0.014 (2)	0.003 (2)	−0.001 (2)
C4	0.043 (2)	0.086 (4)	0.037 (3)	−0.001 (2)	0.0058 (19)	−0.005 (2)
C5	0.052 (3)	0.060 (3)	0.034 (2)	0.010 (2)	−0.0023 (19)	−0.008 (2)
C6	0.049 (2)	0.047 (2)	0.038 (2)	−0.0004 (19)	−0.0029 (19)	−0.005 (2)
C7	0.067 (3)	0.048 (3)	0.085 (4)	−0.017 (2)	−0.015 (3)	−0.009 (3)
C8	0.048 (2)	0.033 (2)	0.035 (2)	0.0020 (18)	−0.0023 (18)	−0.0068 (18)
C9	0.053 (2)	0.030 (2)	0.033 (2)	−0.0063 (18)	0.0004 (18)	−0.0085 (17)
C10	0.049 (2)	0.0258 (19)	0.037 (2)	−0.0038 (17)	−0.0020 (18)	0.0001 (17)
C11	0.058 (3)	0.039 (2)	0.066 (3)	−0.015 (2)	0.011 (2)	−0.016 (2)
C12	0.070 (3)	0.046 (3)	0.068 (3)	−0.009 (2)	0.018 (2)	−0.020 (2)
C13	0.050 (3)	0.047 (3)	0.049 (3)	−0.005 (2)	0.004 (2)	0.007 (2)
C14	0.056 (3)	0.053 (3)	0.046 (3)	−0.020 (2)	−0.008 (2)	0.002 (2)
C15	0.061 (3)	0.041 (2)	0.040 (3)	−0.010 (2)	−0.002 (2)	−0.0085 (19)
C16	0.041 (2)	0.043 (2)	0.031 (2)	−0.0017 (18)	−0.0017 (17)	−0.0009 (19)
C17	0.054 (3)	0.043 (2)	0.046 (3)	−0.001 (2)	−0.004 (2)	−0.002 (2)
C18	0.072 (3)	0.062 (3)	0.047 (3)	−0.002 (2)	0.010 (2)	0.011 (3)
C19	0.077 (3)	0.074 (4)	0.037 (3)	0.007 (3)	0.010 (2)	−0.002 (3)
C20	0.055 (3)	0.058 (3)	0.046 (3)	0.007 (2)	−0.004 (2)	−0.014 (2)
C21	0.049 (2)	0.045 (2)	0.042 (3)	0.0040 (19)	−0.0046 (19)	−0.005 (2)
C22	0.129 (5)	0.054 (3)	0.085 (4)	−0.028 (3)	−0.029 (4)	0.023 (3)
C23	0.045 (2)	0.034 (2)	0.039 (2)	−0.0010 (18)	−0.0025 (18)	−0.0088 (19)
C24	0.042 (2)	0.029 (2)	0.047 (3)	−0.0009 (17)	−0.0016 (18)	−0.0052 (19)
C25	0.039 (2)	0.034 (2)	0.034 (2)	0.0023 (16)	−0.0053 (17)	−0.0058 (18)
C26	0.064 (3)	0.036 (2)	0.050 (3)	−0.010 (2)	0.006 (2)	−0.006 (2)
C27	0.070 (3)	0.050 (3)	0.045 (3)	−0.001 (2)	0.007 (2)	0.003 (2)
C28	0.053 (2)	0.055 (3)	0.030 (2)	0.009 (2)	−0.0051 (19)	−0.007 (2)
C29	0.064 (3)	0.043 (2)	0.050 (3)	−0.001 (2)	−0.006 (2)	−0.019 (2)
C30	0.064 (3)	0.035 (2)	0.036 (2)	−0.0056 (19)	0.0026 (19)	−0.0056 (18)

### Geometric parameters (Å, °)

**Table:**

Br1—C5	1.905 (4)	C10—C15	1.386 (5)
Br2—C20	1.891 (5)	C11—C12	1.390 (6)
Cl1—C13	1.744 (4)	C11—H11	0.9300
Cl2—C28	1.736 (4)	C12—C13	1.367 (6)
O1—C2	1.371 (5)	C12—H12	0.9300
O1—C7	1.424 (5)	C13—C14	1.362 (6)
O2—C9	1.224 (4)	C14—C15	1.372 (6)
O3—C17	1.359 (5)	C14—H14	0.9300
O3—C22	1.419 (5)	C15—H15	0.9300
O4—C24	1.222 (4)	C16—C21	1.372 (5)
N1—C8	1.268 (4)	C16—C17	1.407 (5)
N1—N2	1.369 (4)	C16—C23	1.462 (5)
N2—C9	1.352 (5)	C17—C18	1.379 (6)
N2—H2	0.894 (10)	C18—C19	1.361 (7)
N3—C23	1.281 (5)	C18—H18	0.9300
N3—N4	1.378 (4)	C19—C20	1.378 (6)
N4—C24	1.349 (5)	C19—H19	0.9300
N4—H4A	0.893 (10)	C20—C21	1.385 (6)
C1—C6	1.393 (5)	C21—H21	0.9300
C1—C2	1.397 (5)	C22—H22A	0.9600
C1—C8	1.475 (5)	C22—H22B	0.9600
C2—C3	1.387 (5)	C22—H22C	0.9600
C3—C4	1.367 (6)	C23—H23	0.9300
C3—H3	0.9300	C24—C25	1.498 (5)
C4—C5	1.380 (6)	C25—C30	1.376 (5)
C4—H4	0.9300	C25—C26	1.399 (5)
C5—C6	1.363 (5)	C26—C27	1.356 (6)
C6—H6	0.9300	C26—H26	0.9300
C7—H7A	0.9600	C27—C28	1.375 (6)
C7—H7B	0.9600	C27—H27	0.9300
C7—H7C	0.9600	C28—C29	1.370 (6)
C8—H8	0.9300	C29—C30	1.382 (6)
C9—C10	1.488 (5)	C29—H29	0.9300
C10—C11	1.378 (6)	C30—H30	0.9300
			
C2—O1—C7	118.0 (3)	C13—C14—H14	120.3
C17—O3—C22	119.0 (4)	C15—C14—H14	120.3
C8—N1—N2	116.5 (3)	C14—C15—C10	121.0 (4)
C9—N2—N1	118.4 (3)	C14—C15—H15	119.5
C9—N2—H2	123 (3)	C10—C15—H15	119.5
N1—N2—H2	118 (3)	C21—C16—C17	119.1 (4)
C23—N3—N4	114.4 (3)	C21—C16—C23	122.3 (4)
C24—N4—N3	119.1 (3)	C17—C16—C23	118.6 (4)
C24—N4—H4A	120 (3)	O3—C17—C18	124.6 (4)
N3—N4—H4A	121 (3)	O3—C17—C16	115.6 (4)
C6—C1—C2	118.0 (4)	C18—C17—C16	119.8 (4)
C6—C1—C8	120.1 (3)	C19—C18—C17	120.7 (4)
C2—C1—C8	122.0 (4)	C19—C18—H18	119.7
O1—C2—C3	124.6 (4)	C17—C18—H18	119.7
O1—C2—C1	115.0 (3)	C18—C19—C20	119.9 (5)
C3—C2—C1	120.4 (4)	C18—C19—H19	120.1
C4—C3—C2	120.4 (4)	C20—C19—H19	120.1
C4—C3—H3	119.8	C19—C20—C21	120.5 (4)
C2—C3—H3	119.8	C19—C20—Br2	119.4 (4)
C3—C4—C5	119.5 (4)	C21—C20—Br2	120.1 (3)
C3—C4—H4	120.3	C16—C21—C20	120.1 (4)
C5—C4—H4	120.3	C16—C21—H21	119.9
C6—C5—C4	120.9 (4)	C20—C21—H21	119.9
C6—C5—Br1	119.3 (3)	O3—C22—H22A	109.5
C4—C5—Br1	119.8 (3)	O3—C22—H22B	109.5
C5—C6—C1	120.9 (4)	H22A—C22—H22B	109.5
C5—C6—H6	119.6	O3—C22—H22C	109.5
C1—C6—H6	119.6	H22A—C22—H22C	109.5
O1—C7—H7A	109.5	H22B—C22—H22C	109.5
O1—C7—H7B	109.5	N3—C23—C16	120.4 (4)
H7A—C7—H7B	109.5	N3—C23—H23	119.8
O1—C7—H7C	109.5	C16—C23—H23	119.8
H7A—C7—H7C	109.5	O4—C24—N4	122.8 (4)
H7B—C7—H7C	109.5	O4—C24—C25	121.2 (3)
N1—C8—C1	118.4 (3)	N4—C24—C25	116.0 (3)
N1—C8—H8	120.8	C30—C25—C26	118.5 (4)
C1—C8—H8	120.8	C30—C25—C24	124.5 (3)
O2—C9—N2	122.3 (4)	C26—C25—C24	117.0 (3)
O2—C9—C10	121.5 (3)	C27—C26—C25	120.9 (4)
N2—C9—C10	116.2 (3)	C27—C26—H26	119.6
C11—C10—C15	118.8 (4)	C25—C26—H26	119.6
C11—C10—C9	122.3 (3)	C26—C27—C28	119.7 (4)
C15—C10—C9	118.9 (4)	C26—C27—H27	120.1
C10—C11—C12	120.2 (4)	C28—C27—H27	120.1
C10—C11—H11	119.9	C29—C28—C27	120.9 (4)
C12—C11—H11	119.9	C29—C28—Cl2	119.6 (4)
C13—C12—C11	119.5 (4)	C27—C28—Cl2	119.6 (3)
C13—C12—H12	120.3	C28—C29—C30	119.3 (4)
C11—C12—H12	120.3	C28—C29—H29	120.4
C14—C13—C12	121.1 (4)	C30—C29—H29	120.4
C14—C13—Cl1	119.4 (3)	C25—C30—C29	120.8 (4)
C12—C13—Cl1	119.4 (4)	C25—C30—H30	119.6
C13—C14—C15	119.5 (4)	C29—C30—H30	119.6

### Hydrogen-bond geometry (Å, °)

**Table:**

*D*—H···*A*	*D*—H	H···*A*	*D*···*A*	*D*—H···*A*
N2—H2···O4^i^	0.894 (10)	2.026 (16)	2.900 (4)	165 (4)
N4—H4A···O2^ii^	0.893 (10)	1.994 (18)	2.854 (4)	161 (4)

Symmetry codes: (i) −*x*+1, −*y*, −*z*+1; (ii) −*x*+1, −*y*+1, −*z*+1.
